# D- and L-lactate consumers are taxonomically, biochemically, and energetically different

**DOI:** 10.1093/ismeco/ycag180

**Published:** 2026-06-26

**Authors:** Maximilienne T Allaart, Alexander V Tyakht, Ruth E Ley, Martin Pabst, Gerben R Stouten, Largus T Angenent

**Affiliations:** Environmental Biotechnology Group, Department of Geosciences, University of Tübingen, Schnarrenbergstraße 94-96, 72076 Tübingen, Germany; Department of Microbiome Science, Max Planck Institute for Biology, Max Planck Ring 5, 72076 Tübingen, Germany; Department of Microbiome Science, Max Planck Institute for Biology, Max Planck Ring 5, 72076 Tübingen, Germany; Environmental Biotechnology, Department of Biotechnology, Delft University of Technology, Van der Maasweg 9, 2629 Delft, HZ, The Netherlands; Environmental Biotechnology Group, Department of Geosciences, University of Tübingen, Schnarrenbergstraße 94-96, 72076 Tübingen, Germany; Environmental Biotechnology Group, Department of Geosciences, University of Tübingen, Schnarrenbergstraße 94-96, 72076 Tübingen, Germany; Department of Biological and Chemical Engineering, Aarhus University, Gustav Wieds Vej 10D, 8000 Aarhus C, Denmark; AG Angenent, Max Planck Institute for Biology, Max Planck Ring 5, D-72076 Tübingen, Germany; Cluster of Excellence—Controlling Microbes to Fight Infections, University of Tübingen, Auf der Morgenstelle 28, 72074 Tübingen, Germany; The Novo Nordisk Foundation CO_2_ Research Center (CORC), Aarhus University, Gustav Wieds Vej 10C, 8000 Aarhus C, Denmark

**Keywords:** microbiota, acrylate pathway, methylmalonyl-CoA pathway, propionate, chemostat bioreactor, lactate stereoisomers

## Abstract

D- and L-lactate are routinely produced as intermediates in fermentative ecosystems. However, the microbial fate of these stereoisomers remains poorly understood. Given that D-lactate is an unavoidable byproduct of digestion and a neurotoxin, understanding its microbial turnover not only holds ecological pertinence but also the potential to uncover new links between gut microbiota metabolism and host health. Here, we used chemostat bioreactors (pH 7.0, 37°C, and a solids retention time of 4 days) to enrich for lactate-consuming communities. DL-lactate-consuming consortia were enriched, characterized, and used as inoculum for duplicate bioreactors fed exclusively with D- or L-lactate. After steady-state was reached, the fed lactate stereoisomers were switched to assess community resilience. Regardless of the fed stereoisomer, the fermentation product spectra were consistent and dominated by acetate, propionate, and CO_2_. However, microbial communities and biomass yields diverged sharply, with a high relative abundance of *Anaerotignum* in D-lactate enrichments and *Acidipropionibacterium* and *Propionibacterium* in L-lactate enrichments. Notably, the biomass yield for D-lactate feeding was less than half that for L-lactate feeding, suggesting that the two isomers are metabolized through distinct biochemical pathways despite similar product spectra. Metagenomic and metaproteomic analyses confirmed divergence in D- and L-lactate conversion at both the phylogenetic and pathway levels. Our findings reveal how the stereoisomer identity of microbes shapes their niche specialization, with implications for understanding the ecology and clinical impact of lactate metabolism.

## Introduction

Lactate is a central intermediate in anaerobic ecosystems. Its conversion produces short- and medium-chain carboxylates, such as acetate, propionate, *n*-butyrate, valerate, and caproate [1, 2]. These transformations occur via distinct metabolic routes, including the methylmalonyl-CoA and acrylate pathways for acetate:propionate, and reverse β-oxidation for medium-chain carboxylate production [3, 4]. Although these pathways are well-characterized, the ecological strategies underlying lactate consumption, especially the differential fate of its two stereoisomers, D- and L-lactate, remain poorly understood.

Lactate accumulation is rare in balanced ecosystems, suggesting a tight coupling between lactate producers and consumers [5]. This coupling appears across systems: in ruminant digestive tracts [6, 7], anaerobic digesters [8], soils [9], wetlands ,[10], and the human gut [2, 11], lactate is a transient intermediate, only accumulating under imbalance. Given the transient nature of lactate in balanced ecosystems, following stereoisomer-specific dynamics in situ is challenging due to its rapid turnover, resulting in low steady-state concentrations (<5 mM, [11]). Chemostat bioreactors offer a powerful tool to study conversions at near-zero dissolved concentrations [12, 13], enabling precise control of substrate input while maintaining lactate-limited conditions. These systems enable the mechanistic interrogation of microbial metabolic preferences, resilience, and ecological roles, providing an interesting platform to disentangle lactate isomer metabolism in fermentative ecosystems.

The human gut presents an interesting ecosystem for studying lactate metabolism, as lactate fermentation products are associated with host health benefits [2, 14–16]. Additionally, D-lactate is of particular clinical interest. It is not produced by human cells, only within the gut microbiota, and is linked to neurodegenerative diseases, inflammation, and cancer under dysbiotic or pathophysiological conditions [6, 17–21]. To date, different lactate-consuming taxa have been isolated from fecal samples [3, 14, 22]. Generally, isolation workflows rely on batch cultivation in media containing lactate and various other complex carbon and nitrogen sources. This isolation approach selects for fast-growing microbes that thrive in complex media with high lactate concentrations (30–40 mM, [22]), but not for potentially slower microbes that use lactate as their primary carbon and energy source. Such microbes could play a crucial role in the healthy human gut, where lactate is a limiting substrate, and are currently understudied.

The main research question we address in this study is whether D- and L-lactate are consumed by generalist (consumers of both isomers) or specialist microbes (single-isomer consumers) in lactate-limited conditions, and whether these stereoisomers are metabolized via distinct biochemical and energetic pathways. We employed chemostat bioreactors to enrich and characterize DL-lactate-consuming consortia. Subsequently, the culture was split into bioreactors that were fed exclusively D- or L-lactate to compare fermentation stoichiometry, community composition, and growth kinetics. We identified metabolic specialization and pathway divergence between the two isomer-fed communities using metagenomic and metaproteomic analyses. Our results show that even when end products are conserved, lactate isomers select for distinct microbial taxa and strategies, underscoring the importance of stereochemistry in shaping microbial ecology.

## Materials and methods

### Media composition

The cultivation of gut microbes is often performed in complex media to mimic the complexity of substrates and nutrients that are found in the human gut. The microbes residing in the gut might, therefore, rely on externally available compounds. However, using complex media makes it challenging to disentangle experimental data, because side populations can develop that feed on the complex carbon sources or proteins in gut microbe media. Here, we aimed to enrich lactate consumers without considerable growth of side populations that do not use lactate as main carbon and energy source. Therefore, we designed a selective medium based on previously developed, chemically defined media for gut microbes and for the cultivation of different types of anaerobes [5, 23–26]. We limited the availability of complex nutrients to prevent the development of pertinent side populations, while still aiming to support lactate consumers with auxotrophies. To this end, a B vitamin solution and yeast extract were added to the medium, but they comprised only 5% of the total carbon fed to the bioreactor. The medium contained 100 mM lactate (300 mCmol l^−1^), 50 mM acetate (100 mCmol l^−1^) and 0.5 g yeast extract per liter (assuming a biomass composition of C_1_H_1.8_O_0.5_N_0.2_, this corresponds to 20.3 mCmol l^−1^), amounting to 5% carbon from yeast extract. The full media composition is detailed in the Supplementary Information.

### Bioreactor setup and control

Chemostat cultivation of lactate-consuming microbial communities, which were enriched from the human gut microbiota, was performed in 1.5-l DASGIP parallel bioreactor systems with 1.0-l working volume. The temperature was maintained at 37°C using a DASBOX4 heating block, and the pH was controlled at 7.0 ± 0.1 using 2 M NaOH and 2 M HCl solutions, which were dosed with an MP8 pump module (DASGIP, Germany) that was controlled by custom software [27]. The bioreactors were stirred at 200 rpm using mechanical stirrers with Rushton impellers and continuously sparged with N_2_ gas at 50 ml min^−1^ using an MX4/4 module (DASGIP, Germany) to ensure anaerobic conditions. Pumping influent and effluent to and from the bioreactors was done using custom pump modules (Demo TU Delft, The Netherlands). The off-gas was cooled using a cryostat (Mini-chiller 300, Huber, Germany), which was set to 5°C to prevent evaporation of the culture broth.

### Experiment I: Chemostat cultures fed with DL-lactate

To start the bioreactor, the inoculum (Supplementary Information) was added to 1 l of fresh medium, which was identical to that used in the anaerobic serum bottles. The bioreactor was then operated in batch mode. Considerable microbial activity was only observed after ~30 days of incubation. This was likely due to low cell numbers of lactate consumers in the inoculum and adaptation to bioreactor conditions and selective lactate medium. The gassing conditions (headspace vs. liquid gassing) and stirring speed were adjusted during the initial batch phase to accommodate better growth. When consistent microbial activity occurred, the bioreactor was stirred at 200 rpm with liquid gassing. After lactate was depleted in the initial batch phase, 50% of the bioreactor volume was exchanged with fresh medium, upon which direct activity was observed. Next, lactate was depleted within 24 h after which the bioreactor was switched to chemostat mode at a dilution rate (*D*) of 0.01 h^−1^.

After 10 days of operation in chemostat mode (i.e. constant feeding and mixing), the effluent of the bioreactor was used to inoculate a second bioreactor, which was operated in identical conditions, serving as a biological replicate. Both bioreactors were initially operated at a solids retention time (SRT) of 96 h (*D* = 0.01 h^−1^). This retention time falls within the range of measured gut transit times in humans, specifically slow transit times. It was shown that slow transit times correlate to the highest microbiota diversity, indicating that longer residence times in the bioreactor are likely to support the most diverse range of microbes to thrive [28]. Additionally, transit time and microbial residence are not identical, as gut microbiota can increase their residence time by residing in biofilms, further rationalizing the choice for an SRT corresponding to slow transit times. No considerable biofilm formation on the glass and other surfaces within the bioreactors was observed during the operation of the bioreactors, confirming that our hydraulic retention time (HRT) and SRT were the same. Steady state was assumed when the main product concentrations in the bioreactors and the gases in the bioreactor off-gas fluctuated <10% for at least 3 SRTs (Replicate I) or 2 SRTs (Replicate II). After steady state was maintained for 2 (Replicate II) or 3 (Replicate I) SRTs, the dilution rate in the chemostat was doubled. This amounted to an SRT of 48 h (*D* = 0.02 h^−1^). The deviation in Replicate II arose from experimental time constraints, which necessitated changing the dilution rate in this replicate after two SRTs rather than three. However, system performance metrics had already stabilized by this point and were highly comparable to the physiology observed in Replicate I, confirming stable operation and reproducibility. At the end of the enrichment, the contents of the reactors were mixed and stored at 4°C. Biomass concentrations and bioreactor- and biomass-specific rates were calculated from the steady state data, and the standard deviations in these kinetic parameters were calculated using error propagation with the uncertainties package in Python.

### Experiment II: Chemostat cultures fed with exclusively D- or L-lactate

Two single chemostat bioreactors that were fed exclusively with D- or L-lactate were inoculated with the stored broth from Experiment I, which harbored a population of DL-lactate-consuming microbes. One liter of batch medium, containing only D- or L-lactate, was inoculated with 100 ml of stored broth in the two separate bioreactors. The initial batch phase took approximately 10 days for D-lactate and approximately 4 days for L-lactate. After this initial batch phase, the chemostat mode was initiated at *D* = 0.01 h^−1^, and the effluent from the bioreactors was used to inoculate the biological duplicates for each of the two bioreactors, resulting in four bioreactors. The biological replicates contained 500 ml of fresh medium and were operated in continuous mode directly (i.e. effluent from the first replicate bioreactor and media at *D* = 0.01 h^−1^ were fed simultaneously until the working volume of 1 l was reached). Steady state was assumed when the main product concentrations in the bioreactors and the gases in the bioreactor off-gas fluctuated <10% for at least 3 SRTs; biological rates and their uncertainties were calculated as described for Experiment I. To calculate the statistical significance of the difference in biomass concentration between exclusive D- and L-lactate feeding, we performed a Welch’s *t-*test, which is suitable for systems with unequal variances and sample sizes.

### Analytical methods

The biomass concentration in the bioreactor was monitored with the OD_600_ by using a spectrophotometer (BioMateTM160, Thermo Scientific, United States). Cell dry weight was measured by taking 15 ml of culture broth, centrifuging, washing the pellet with MQ water, and then freeze-drying the pellet. The freeze-dried pellets were weighed, and the biomass concentration was correlated to the measured OD_600_. The resulting conversion factor from OD_600_ to biomass concentration (gDW/l) was 0.298, which was consistent among both D- and L-lactate and used for all enrichments (Supplementary Fig. S1). No significant difference in conversion factors or bacterial morphology was observed between D- and L-lactate communities within the experimental OD range; therefore, a unified factor was adopted. All biomass measurements were performed in duplicate. Furthermore, the culture was inspected routinely using a microscope (Olympus BX41, Japan) to monitor for morphological changes. Samples of the culture broth were centrifuged (Eppendorf, Germany) for 5 min at 14,000 rpm (~18 500 RCF) and room temperature, and the supernatant was filtered through a 0.22 μm PVDF syringe filter (Carl Roth, Germany). Pellets and supernatants were stored separately at −20°C until further use. Organic acids were measured from the supernatant samples with high-performance liquid chromatography (HPLC), using an Aminex HPX-87H column at 60°C coupled to a UV–VIS detector at 210 nm (Shimadzu, Germany). 5 mM sulfuric acid was used as eluent. Total lactate was quantified using HPLC, and individual isomers were quantified using a spectrophotometric D/L-lactate rapid assay kit (Megazyme, Neogen, Ireland) following the manufacturer’s instructions. H_2_ and CO_2_ production in the bioreactors was measured using mass spectrometry (Prima BT Benchtop, Thermo Fisher Scientific UK), with N_2_ serving as the inert gas for gas mass balancing.

### Microbial community profiling

The microbial community composition of the bioreactors was analyzed using cell pellets from 3- or 4-ml samples taken at various time points during the enrichments. The initial microbiota composition was analyzed directly from the mouse excrement (Supplementary Fig. S7). DNA was extracted from the cell pellets or murine feces using the AllPrep PowerFecal Pro DNA/RNA extraction kit (Qiagen Inc., Germany) following the manufacturer’s instructions. The DNA content of the extracts was quantified using a Qubit 4 (Thermo Fisher Scientific, USA) to confirm that sufficient DNA was extracted for analysis. Additionally, samples from the D- and L-lactate enrichments immediately before and after the substrate switch (*n* = 8) were used for shotgun metagenomic sequencing. Sequencing and data processing methods for amplicon and metagenomic sequencing and methods for investigation of lactate utilization pathways are detailed in the supplementary material and supplementary Table 1. The sequencing data have been deposited in the European Nucleotide Archive (ENA), project accession PRJEB89830.

### Metaproteomics

Cell pellets from 50 ml culture broth were collected from each biological duplicate after the chemostat conditions were stopped at the end of the D- and L-lactate enrichments. Protein extraction from these cell pellets and metaproteomic analysis were performed as described previously [29, 30] or as further elaborated in the supplementary information. Raw data were searched using PEAKS Studio X (Bioinformatics Solutions Inc., Canada) against protein sequences derived from assembled contigs from whole metagenome sequencing, meaning that each protein in the metaproteome was assigned to a corresponding metagenome-assembled genome (MAG). Search parameters included a 20 ppm parent ion tolerance, 0.02 m/z fragment ion tolerance, allowance for up to two missed cleavages, carbamidomethylation as a fixed modification, and methionine oxidation and N/Q deamidation as variable modifications. A 1% false discovery rate was applied to peptide-spectrum matches, and proteins were considered confidently identified if ≥2 unique peptides were matched. Label-free quantification was performed using PEAKS Q (Bioinformatics Solutions Inc., Waterloo, Canada), with peak areas normalized to the total ion current of each run, allowing 15 ppm mass error and 7.5 min retention time variation across the dataset. We screened the proteomes of the four most abundant MAGs in our dataset (*Anaerotignum propionicum, Acidipropionibacterium jensenii, Propionibacterium freudenreichii,* and an unclassified *Spiro-02* species, see Supplementary Information) for the proteins of interest from the acrylate and methylmalonyl-CoA pathways, resulting in a qualitative description of the species-specific proteome. Protein abundance per sample in each MAG was calculated by dividing the sample area of the protein by the total area of all proteins in that sample. Proteomics data have been deposited in the PRIDE database and can be found under accession number PXD068334.

## Results

### Lactate isomer-consuming enrichment cultures grow in chemostat bioreactors

**Figure 1:**
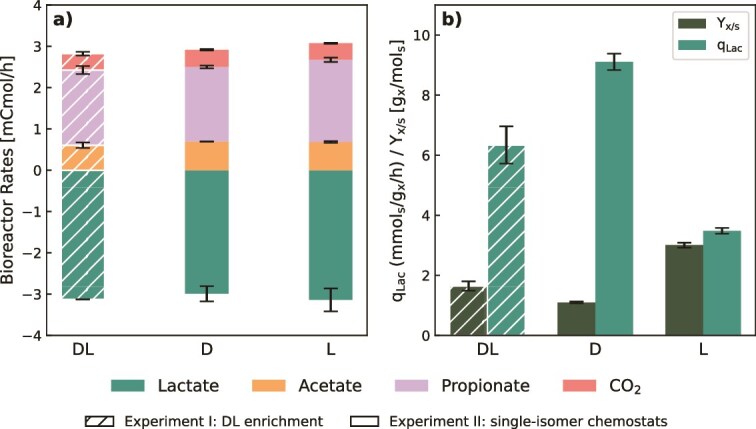
Kinetic parameters during steady-state fermentation of lactate stereoisomers in chemostats. Panel (a) shows the average (mean ± standard deviation) bioreactor-specific conversion rates of lactate and the main fermentation products during DL- (Experiment I), D-, and L- (Experiment II) lactate fermentation in steady-state chemostats in two biological duplicates (i.e. n = 2 for all three conditions). This panel also represents the carbon balance, which was consistently within 90–110% during steady-state, confirming that all main fermentation products and substrates have been identified. Panel (b) shows the average (mean ± SD) biomass yield on lactate (*Y*_x/s_) for each substrate, and the respective biomass-specific lactate conversion rates (*q*_Lac_).

**Figure 2:**
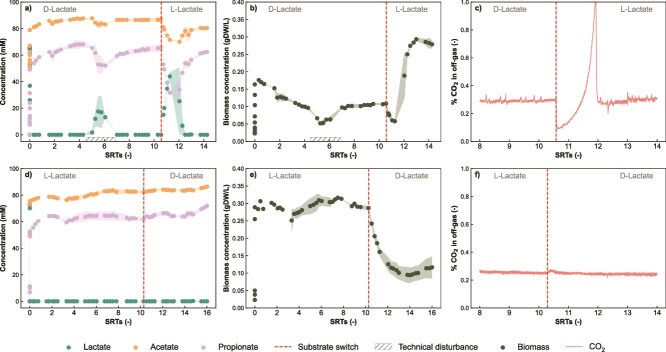
Fermentation profiles of D- and L-lactate for Experiment II chemostat bioreactors and their response to substrate switch. Panels (a and d) Product concentrations and panels (b and e) biomass concentrations in biological replicate bioreactors initially fed with D-lactate (top row, *n* = 2) and L-lactate (bottom row, *n* = 2) and switched to the other isomer, indicated with the dashed lines. Panels (c and f) show the high-resolution response to the substrate switch, as observed from the CO_2_ profiles measured with off-gas mass spectrometry. Full gas profiles of all bioreactors are presented in Supplementary Fig. S4. Duplicate bioreactors were started from the initial chemostat reactors after 1.5 and 2.5 SRTs for D-lactate and L-lactate, respectively. The closed circles represent the mean of the biological replicates, and the shaded area represents the half range (i.e. the distance between the measured data points and the mean). Both bioreactors initially fed with D-lactate suffered from a technical disturbance (clogged feed line) around 5 SRT periods, causing the spike in lactate concentration and dips in acetate, propionate, and biomass in panels (a and b), and recovered within 2 SRTs.

We performed two subsequent bioreactor experiments, comprising three different feeding conditions, to study lactate isomer metabolism in lactate-limited conditions. For Experiment I, a DL-lactate-consuming microbial community was enriched from humanized mouse feces in duplicate chemostat bioreactors under gut-relevant conditions (pH 7.0, 37°C, dilution rate of 0.01 h^−1^). We characterized the steady-state fermentation parameters of the culture during Experiment I and subsequently inoculated two separate sets of duplicate bioreactors with the DL-culture, feeding them exclusively D- or L-lactate, respectively (Experiment II). Across both experiments and independently of the fed isomer, the steady-state fermentation stoichiometries were similar: acetate, propionate, and CO_2_ were consistently produced in a 1:2:1 molar ratio (Figs 1 and 2, Supplementary Fig. S2).

**Figure 3:**
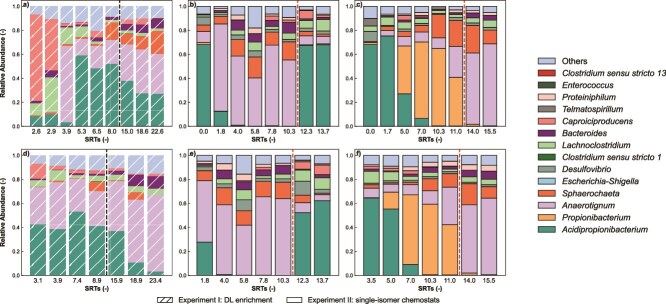
Microbial community composition development for Experiments I and II. 16S rRNA gene amplicon sequencing for community profiling in DL-lactate-fed chemostat bioreactors (Experiment I, *n* = 2) and exclusively D-, and L-lactate-fed bioreactors (Experiment II, *n* = 2 for each isomer) throughout time. Each panel represents an individual biological replicate. For Experiment I, the DL bioreactors (a and d) went through an increase in dilution rate from 0.01 to 0.02 h^−1^, which is indicated with the dashed line. The SRTs at the sampling points for Experiment I were not equal because one of the bioreactors was temporarily kept at a lower dilution rate before reaching steady state to allow recovery from a technical disturbance (Supplementary Fig. S2). Single-isomer fed bioreactors for Experiment II (b and e: D-lactate; c, f: L-lactate) went through a substrate switch, which is indicated with the dashed line.

Despite this metabolic convergence, community structures profiled with 16S rRNA gene amplicon sequencing diverged markedly. DL-lactate enrichments (Experiment I) were dominated by *Anaerotignum* and *Acidipropionibacterium* (Fig. 3a and d). For Experiment II, D-lactate-fed chemostats selected strongly for *Anaerotignum* (Fig. 3b and e), whereas L-lactate-fed chemostats initially favored *Acidipropionibacterium* before shifting toward *Propionibacterium* dominance (Fig. 3c and f). Under all conditions, *Sphaerochaeta, Bacteroides,* and *Lachnoclostridium* were part of the community, albeit with lower relative abundances compared to the main community members. Shotgun metagenomic sequencing followed by assembly and binning identified the dominant taxa as *A. propionicum, A. jensenii, P. freudenreichii, Spiro-02 sp001604275*, and *Bacteroides thetaiotaomicron* (Supplementary Fig. S3)*.*

### D- and L-lactate consumers differ in biomass yield and kinetics despite identical product spectra

To gain a deeper understanding of the physiology of the isomer-consuming microbes, we evaluated their growth kinetics. Although main product ratios were invariant, the average (mean ± SD) steady-state biomass yields differed threefold between isomers: 1.10 ± 0.03 g_x_ mol_lactate_^−1^ for D-lactate versus 3.01 ± 0.08 g_x_ mol_lactate_^−1^ for L-lactate (Fig. 1b). This discrepancy is unexpected, given identical dilution rates and input concentrations, suggesting the existence of distinct energy-conserving pathways. Biomass-specific lactate uptake rates were inversely correlated with yield: three-fold higher for D-lactate consumers than for L-lactate consumers (Fig. 1b). These contrasting strategies are highly significant (*P* < <0.001, Welch’s *t-*test, *t* = 54.6, *P* = 6 × 10^−36^; *n* = 18 for D-lactate, *n* = 20 for L-lactate) and likely alter competitive dynamics in lactate-limited systems. The affinity for substrate is a key parameter in chemostat systems, and microbes with a high affinity for the limiting substrate usually thrive. However, because the D-lactate consumers have a high biomass-specific lactate conversion rate, competition based on substrate uptake rate cannot be excluded in this system.

### Substrate switching reshapes community structure and activity

Next, we investigated the response to switching the lactate isomer in the bioreactor feed within Experiment II (Fig. 2). After maintaining steady-state conditions for at least 3 SRTs and characterizing the fermentation parameters, the lactate isomer in the bioreactor feed was switched: the D-lactate-fed bioreactors received only L-lactate, and vice versa. The off-gas profile (Fig. 2c and f) is a real-time reflection of the microbial activity in the bioreactor [27] and the constant presence of ~0.3% CO_2_ in the off-gas corresponded to the continuous conversion of lactate for the continuously fed bioreactors. The horizontal profiles of the figures after 2–3 SRTs are a hallmark of steady-state chemostat operation, corresponding to stable product and biomass concentrations (Fig. 2a, b, d, and  f).

Switching from D- to L-lactate resulted in an immediate decline in microbial activity, as evidenced by lactate accumulation and a fast decrease in the CO_2_ concentration in the off-gas (Fig. 2a and c). Recovery involved exponential growth and a shift from *Anaerotignum* to *Acidipropionibacterium* dominance (Figs 2c and 3b and e). Following this exponential growth, the biomass concentration stabilized at approximately 0.28 g/l (Fig. 2b), which is comparable to the biomass concentration (0.30 ± 0.008 g/l) *prior* to the substrate switch in the L-lactate enrichment (Fig. 2e). Meanwhile, the concentrations of CO_2_, lactate, and product returned to steady-state values (Fig. 2a). Conversely, switching from L- to D-lactate caused minor perturbation in the CO_2_ profile or product spectrum (Fig. 2d and f). However, the biomass concentration gradually decreased to ±0.13 g/l (Fig. 2e), and *Propionibacterium* was replaced by *Anaerotignum* as the predominant genus.

### Combined metagenomics and proteomics reveal isomer-specific metabolic pathways

The combined physiological and community data led us to hypothesize that D- and L-lactate-consuming microbes must employ distinct biochemical pathways to account for the significant change in biomass concentration. To address this question, we combined metagenomics and metaproteomics of the community before and after the substrate switch. The relative abundance of the *Anaerotignum, Acidipropionibacterium, Propionibacterium*, and *Spiro-02* MAGs and metaproteomes corresponded well to the relative abundance measured with 16S rRNA gene amplicon sequencing (Fig. 3, Supplementary Fig. S3). A single, representative high-quality MAG was identified for each of these genera. No other MAGs had a high relative abundance across all conditions.

**Figure 4:**
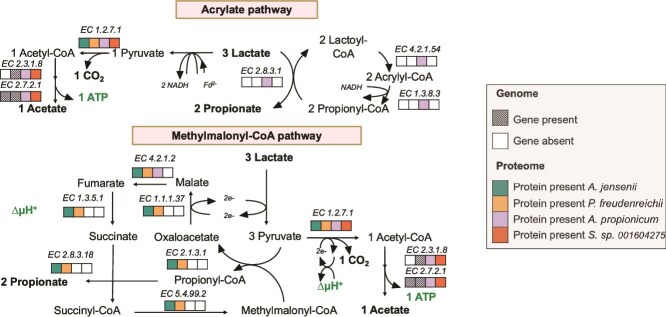
Metagenome and metaproteome analysis of Experiment II. The metaproteome of the communities was analyzed at the end of the bioreactor runs. The metagenomes provided the search database for protein identification. We identified the pathway enzymes required for the acrylate and methylmalonyl-CoA pathways based on the KEGG and Uniprot databases and separated the metaproteomics data into the proteins pertaining to our four main microbial species. Then, we searched the species’ MAGs and proteomes for the enzymes involved in the acrylate and methylmalonyl-CoA pathways. Presence of both gene and protein is indicated with a colored box, presence of the gene with a patterned grey box, and absence of both gene and protein with a white box.

Mapping the proteome to the metagenomic database (Fig. 4) shows that the *A. jensenii* and *P. freudenreichii* MAGs and proteomes contain the key enzymes required for the methylmalonyl-CoA pathway. *A. propionicum*, on the other hand, does not possess the genes for the methylmalonyl-CoA pathway but instead employs the acrylate pathway for lactate utilization. *A. propionicum* was the main D-lactate-consuming taxon, and interestingly, the conversion of lactoyl-CoA to acrylyl-CoA is D-lactoyl-CoA-specific [31, 32]. This explains the isomer preference of this microbe. *Spiro-02 sp001604275* does not possess either of these pathways. Additional analysis of the lactate conversion pathways, as described previously [3], shows no involvement of *Spiro-02 sp001604275* in *n*-butyrate or 1,2-propanediol production either and confirms the use of the methylmalonyl-CoA pathway by *A. jensenii* and *P. freudenreichii* and the acrylate pathway by *A. propionicum* (Supplementary Fig. S5)*.* These two pathways are energetically distinct, with the acrylate pathway yielding one ATP per 3 mol of lactate, and the methylmalonyl-CoA pathway yielding at least 2^1^/_3_ ATP per 3 mol of lactate [33]. This energetic difference explains the disparity in biomass yield between D- and L-lactate (Fig. 1b).

### Proteomic insights into energy conservation and accessory functions

To verify the energetics of the two distinct lactate utilization pathways in our microbial communities, we utilized our meta-omics data to identify the enzymes involved in lactate transport and utilization. Gene annotations of our MAGs suggest that lactate is permeated by an L-lactate permease or a glycolate transporter, which transport both D- and L-lactate despite their annotation [34]. The identification of lactate utilization genes using marker Pfam domains (see Supplementary Methods) highlighted multiple loci of colocalization, providing additional evidence that many of these genes act in a co-regulated manner (Supplementary Fig. S6). The cofactors used in converting lactate to pyruvate dictate the downstream pathway biochemistry, and these first enzymes are distinct in the acrylate and methylmalonyl-CoA pathways. The *lutABC* operon enables L-lactate utilization, linking it to cytochrome reduction and energy generation through the electron transport chain. Our *A. jensenii* and *P. freudenreichii* MAGs and proteomes encode these lactate utilization proteins LutABC. For D-lactate utilization, it is likely that an electron-confurcating LDH, such as LctBCD in *Acetobacterium woodii* [35]*,* is employed by *A. propionicum.* Indeed, the EtfA and EtfB proteins (otherwise referred to as LctB and LctC) required to form the confurcating complex are abundantly present in the *A. propionicum* proteome. LctD was not found in the genome, but we identified another lactate utilization protein (FOHMDGFI_02069) [36] after refining our MAGs annotation with PGAP, which could exhibit the function of LctD. NAD-dependent D- and L-LDH (ECs 1.1.1.27 and 1.1.1.28) were also found to be encoded in the bacterial genomes from our enrichment cultures across all conditions, but have been shown to preferentially confer lactate production rather than utilization capacities [37–39].

Because the physiological profiles and meta-omics signatures for the D- and L-lactate cultures were internally consistent and clearly distinct, we examined the proteomes for additional features that might contribute to the isomer-specific phenotypes. The most conspicuous signal was in *A. propionicum*: a single, putative tungstate ABC transporter accounted for the highest fraction of total protein; between 10% and 18% across samples, hinting at a tungsten-dependent redox route unique to this taxon. Furthermore, no dominant lactate consumers encoded a lactate racemase, underscoring their specialization for a single stereoisomer. Only the *Spiro-02 sp001604275* MAG carried a racemase gene, giving it the genetic capacity to interconvert D- and L-lactate; however, downstream pathway markers were either absent or below detection, so its role in lactate catabolism remains unresolved.

## Discussion

By coupling long-term chemostat cultivation with metagenomics and metaproteomics, we uncovered two functionally distinct guilds of lactate consumers both forming acetate, propionate and CO_2_: the D-lactate specialist *A. propionicum* and L-lactate specialists *A. jensenii* and *P. freudenreichii.* Metabolic reconstruction and proteomics show that D-lactate is metabolized via the acrylate pathway, whereas L-lactate is metabolized via the methylmalonyl-CoA pathway.

The import of lactate appears to be similar for both stereoisomers; however, the initial lactate-converting enzymes and their downstream biochemistry diverge, resulting in a threefold difference in biomass-specific lactate conversion rates and biomass yields between the isomers. These characteristics suggest that rate versus yield trade-offs might drive the competitive strategies of lactate stereoisomer consumers [40], with D-lactate consumers gaining an advantage from high rates and L-lactate consumers gaining an advantage from high yields. The unusually large tungstate-transporting proteome fraction in *A. propionicum* supports recent reports implicating W-containing electron bi- or confurcating enzymes, hinting at potential additional energetic advantages for the D-lactate pathway [41].

The discovery of distinct biochemical pathways with different kinetic properties for D- and L-lactate conversion is interesting. The main fermentative routes of lactate are the methylmalonyl-CoA pathway (0.78 ATP/mol lactate), the acrylate pathway (0.33 ATP/mol lactate), and reversed β-oxidation (0.5 ATP/mol lactate) [4, 33]. From an energetics point of view, exploiting the methylmalonyl-CoA pathway, which we observed in the L-lactate enrichments, is the most favorable scenario in an affinity-based enrichment such as in a chemostat bioreactor. However, our data suggest that *A. propionicum* gained a competitive advantage in the D-lactate enrichments compared to microbes with higher ATP yields by optimizing its rate [33]. This shows that competition in our chemostat systems depended on both substrate affinity and substrate conversion rate. Chain-elongated products were only detected in concentrations an order of magnitude lower than acetate and propionate, confirming that chain elongation was not a favorable strategy in these conditions. Chain elongation is biochemically more complex, potentially giving *A. propionicum,* employing the relatively simple acrylate pathway, a rate benefit over chain elongators as well as propionate producers employing the methylmalonyl-CoA pathway [29, 40]. Additionally, propionate producers tend to flourish compared to chain elongators at neutral pH [42, 43].

The absence of previously identified major gut lactate consumers (i.e. microbes from the genera *Anaerobutyricum, Anaerostipes*, and *Coprococcus* [14, 44, 45]) is surprising. However, the conditions in our bioreactor systems favored microbes that use lactate as their main carbon and energy source over multi-substrate consumers that require complex nutrients as co-substrates, such as the currently known gut lactate-consuming taxa [46, 47]. This could explain why they did not flourish in our chemostats. *Anaerotignum, Propionibacterium*, and *Acidipropionibacterium* are rarely identified as prevalent taxa in the human gut, but they do find their origins in dairy products and the gut [48–52]. This suggests that they persist in the gut microbiota at low abundance, as is the case for the lactate-consumer functional guild in general [2]. Commonly used gut microbiota composition analyses are biased towards detecting highly abundant species [53], making it entirely possible that genera or species crucial in the conversion of lactate are systematically overlooked. Chemostat enrichments are a powerful tool for enriching and identifying low-abundant functional groups, underscoring the importance of diverse cultivation methods to obtain a complete picture of a complex fermentative ecosystem like the human gut.

From the host perspective, a minority population that removes D-lactate quickly is desirable to prevent accumulation to detrimental levels. Our substrate-switch experiments demonstrated that a small population of D-lactate consumers effectively coped with a rapid influx of D-lactate, as reflected by the lack of lactate accumulation after switching from L- to D-lactate. This phenomenon was not observed for a culture enriched on D-lactate that switched to L-lactate. Whether the host actively maintains such D-lactate specialists (e.g. via micronutrient provisioning) or sustains them through cross-feeding dynamics within the microbiota remains an open question. Understanding these host–microbe interactions could inform interventions for D-lactic acidosis and potentially obesity, which was recently linked to increased circulating D-lactate levels [54].

Our data suggest that our bioreactor systems did not favor racemase-based lactate consumption (i.e. lactate generalist). In substrate-limited chemostats, an equilibrium-based racemase [55] would halve the available concentration of the preferred isomer, and thus depress uptake rates, which is a probable competitive disadvantage compared to single-isomer specialists. By contrast, racemase-positive taxa are enriched in glucose-fed sequencing batch bioreactors that experience high, sustained lactate concentrations, aligning with the idea that racemization is advantageous only when substrate is abundant [5]. *Spiro-02 sp001604275* is the only racemase-encoding taxon in our communities, and the taxon does not gain dominance or encode any known lactate-converting pathways. This indicates that its growth is limited, making it unlikely that it relies solely on lactate as its primary carbon source. We, therefore, hypothesize that *Spiro-02 sp001604275* ferments protein from yeast extract, but our data is not conclusive in resolving its role in the community. A remarkable notion is the presence of *A. propionicum* with low relative abundance in the L-lactate enrichments. This observation leads to the hypothesis that *Spiro-02 sp001604275* might convert a small fraction of the L-lactate to D-lactate, thereby sustaining *A. propionicum.* In this way, a small racemase-expressing population might help sustain a population of D-lactate consumers in the human gut to maintain overcapacity for D-lactate consumption.

More broadly, nature’s strong tendency toward homochirality (e.g. D-sugars in DNA and L-amino acids in proteins [56]) makes the coexistence of both lactate isomers in fermentative ecosystems particularly intriguing. We propose that isomer-specific metabolism may confer competitive advantages to microbes that specialize in one form and enhance ecosystem stability. On an ecosystem scale, metabolic diversity, through the presence of both D- and L-lactate-utilizing taxa, contributes to greater resilience [57] by expanding the available number of niches. Such diversity arises not only from taxonomic variation (e.g. *Anaerotignum* vs. *Acidipropionibacterium* and *Propionibacterium*) but also from distinct metabolic pathways (acrylate vs. methylmalonyl-CoA) and substrate preferences (D- vs. L-lactate), collectively enriching the system’s functional capacity in all aspects of ecosystem diversity: species richness, genetic variation, and functional diversity [58, 59]. We further hypothesize that specialization on different isomers fosters synergistic interactions between lactate producers and consumers [60], including potential exchanges of essential amino acids or vitamins. Complementary biosynthetic capabilities and auxotrophies could reduce individual proteome burdens, leading to higher shared growth efficiencies [61, 62].

## Conclusions

Our work shows that a single microbiota diverged into taxonomically, biochemically, and kinetically distinct microbial populations in D- and L-lactate-fed chemostats. These specialist taxa employed different strategies for converting their preferred lactate isomer to acetate, propionate, and CO_2_. D-lactate was converted through the acrylate pathway at higher biomass-specific rates, while L-lactate was converted through the methylmalonyl-CoA pathway, leading to a higher biomass yield. The isomer-dependent, distinct phenotype developed consistently and was replicated when the isomer in the feed was swapped. Lactate consumers comprise a small fraction of the gut microbiome, and we enriched microbial taxa that are not commonly found among lactate consumers in the gut. This suggests that cultivation-independent approaches may overlook important gut microbial species and that chemostat enrichments offer a valuable tool for studying underrepresented taxa in greater detail. The role of D- and L-lactate consumers in human health and disease provides an interesting avenue for future study.

## Supplementary Material

Supplementary_material_ycag180

## Data Availability

The microbiota sequencing data have been deposited in the European Nucleotide Archive (ENA), project accession PRJEB89830. The essential data analysis code is provided at https://github.com/leylabmpi/dl_lac_react_pub. Proteomics have been deposited in the PRIDE database and can be found under accession number PXD068334.
