# Supplementary material for: D- and L-lactate consumers are taxonomically, biochemically, and energetically different
Source: ISME Commun. 2026 Jun 26;6(1):ycag180. doi: 10.1093/ismeco/ycag180 (PMC13411273; doi:10.1093/ismeco/ycag180)
Supplement: Supplementary_material_ycag180 [file supplementary_material_ycag180.zip › Supplementary_Information_v8_ycag180.docx]

**Supplementary Information**

**Methods**

*Media composition*

The composition per liter of medium was as follows: lactate 9 g, acetate 3 g, yeast extract 0.5 g, NH_4_CL 1.5 g, KH_2_PO_4_ 0.75 g, NaCl 0.5 g, Na_2_SO_4_ 0.088 g, 1 mL trace element solution (100x), 0.1 mL vitamin solution (1000x). The trace element solution (100x) contained (g L^-1^): MgSO_4_ · 7 H_2_O 3.6; MgCl_2_ 0.2; FeSO_4_ · 7 H_2_O 0.1; CaCl_2_ 0.9; MnSO_4_ * H_2_O 0.3; ZnSO_4_ * 7 H_2_O 0.1; CoCl_2_ · 6 H_2_O 0.085; CuCl_2_ · 2 H_2_O 0.068; Na_2_MoO_4_ · 2 H_2_O 0.02; NiCl_2_ · 6H_2_O 0.02; Na_2_SeO_3_ 0.02; Na_2_WO_4_ 0.02. The vitamin solution (1000x) contained (g L^-1^): biotin (B7) 1, nicotinic acid (B3) 0.5, pABA (B10) 0.5, thiamine hydrochloride (B1) 0.5, Ca-pantothenate (B5) 0.5, pyridoxine hydrochloride (B6) 0.5, cyanocobalamin (B12) 0.1, riboflavin (B2) 0.5, folic acid (B9) 0.2, lipoic acid 0.5.

*Inoculum*

Fecal samples from mice colonized with a human gut microbial sample was used for inoculation of the DL-lactate enrichment [1, 2]. Specifically, two fecal pellets (~1-3 mg each) were used, one from a female and one from a male humanized mouse. The fecal samples were stored at -80°C after collection. 16S rRNA gene amplicon sequencing (Supplementary Figure 7) showed that the community composition of the inoculum, with *Bacteriodota,* and *Firmicutes* as the dominant phyla, is comparable to compositions observed in large cohort human microbiome studies, confirming that this is a relevant inoculum to study [2, 3]. Pre-cultures were prepared from the stored feces at 37°C in 100 mL anaerobic serum bottles containing 50 mL of the media described above but with reduced DL-lactate and acetate concentrations of 30 mM and 10 mM, respectively, and including 0.5 g L^-1^ L-cysteine and 0.5 mg L^-1^ resazurin, and N_2_ gas in the headspace. The substrate concentrations were adjusted compared to the bioreactor medium to allow inoculum acclimatization to lactate and to ensure fully anaerobic condition in absence of continuous nitrogen sparging. The fecal pellets were directly inoculated from the freezer, ensuring that the full microbial diversity of the inoculum was present in the pre-culture. After growth was observed, the culture was passed to new serum bottles containing fresh medium. 10 mL of the grown culture from these bottles was used to inoculate Experiment I (see below).

*Amplicon sequencing*

The samples were sent to Novogene Ltd. (Munich, Germany) for amplicon sequencing of the V3-V4 region of the 16S rRNA gene (position 341-806, forward primer sequence CCTAYGGGRBGCASCAG, reverse primer sequence GGACTACNNGGGTATCTAAT) on an Illumina NovaSeq paired-end platform; the resulting coverage was 64,000 ± 3,000 read pairs. The read processing included the following steps (software/databases): adaptor removal (cutadapt) [4]; paired merging (FLASH) [5]; quality filtering (fastp) [6]; chimera removal (vsearch [7] using SILVA database [4, 8]); denoising and ASV generation using DADA2 [9]; and taxonomic classification of ASVs in QIIME2 [10].

## *Metagenomic sequencing and data processing*

Shotgun metagenomic sequencing was then performed on the Illumina NovaSeq platform in 250 bp paired-end format. The genomic DNA was randomly sheared into shorter fragments, and the obtained fragments were then end-repaired, A-tailed, and further ligated with Illumina adapters. The resulting fragments with adapters were size-selected, PCR amplified, and purified. The library was quantified through Qubit and qPCR, and the size distribution was detected with a fragment analyzer. Quantified libraries were pooled and sequenced on Illumina platforms according to the effective library concentration and the required data amount. The raw reads were filtered by removing the reads containing adapters, reads with >10% undetermined bases, and reads with a low-quality score (<50% certainty of correct base calling). Shotgun metagenomic sequencing yielded 48 ± 8 Gbp paired-read coverage per sample.

Preprocessing of the raw metagenomic reads, assembly of metagenomes, generation of metagenome-assembled genomes (MAGs), and taxonomic classification of the MAGs were performed using an in-house pipeline [11]. Per-MAG gene prediction and initial functional annotation were performed using Prokka [12] and eggNOG-mapper [13]. The produced gene sequences were combined across the 102 initial MAGs to be used for the reference database in proteomic experiments. The MAGs were dereplicated at 95% ANI similarity and filtered by quality using dRep [14] to obtain 29 species-representative genomes (SRGs). These were subsequently placed into KrakenUniq [15] to prepare a reference database and quantify the species’ relative abundance in each metagenome. For the initial rough assessment of lactate utilization pathways that were encoded in the bacterial genomes, we prepared two lists of EC gene accessions corresponding to the essential reactions of each of the acrylate and methylmalonyl-CoA pathways for lactate utilization (n = 5 and 9 accessions, respectively). Their presence in each genome was determined based on the eggNOG-mapper-derived annotation described above. For the four MAGs representing the selected species of interest (*Anaerotignum propionicum*, *Acidipropionibacterium jensenii*, *Propionibacterium freudenreichii,* and an unclassified *Spiro-02* species), to broaden the insight into possible lactate utilization-related functions of the genes, in addition to Prokka, the gene annotation was conducted using the BV-BRC portal [16] as well as the stand-alone NCBI Prokaryotic Genome Annotation Pipeline tool [17]. The three annotation tracks were subject to a fuzzy joint (allowing for a maximum of 50 bp discrepancy of the gene start and end coordinates) and summarized for the reference purposes into Supplementary Table 1. Using the Prokka gene annotation and generally following the description provided previously [18], we identified lactate-utilization gene loci based on the presence of Pfam domains using hmmsearch in HMMER3. We also identified marker genes for *n*-butyrate and propionate-producing pathways and Rnf complexes *via* DIAMOND alignment [19]. Supplementary Table 1 (supplied as a separate excel file) contains gene annotations for a) *Acidipropionibacterium jensenii*. b) *Anaerotignum propionicum*. c) *Propionibacterium freudenreichii*. d) Unclassified *Spiro-02* species, combined across Prokka, BV-BRC and PGAP based on the “fuzzy join” with tolerance threshold of 50 bp.

*Metaproteomics*

Sample aliquots corresponding to approximately 250 ng of proteolytic digest were analyzed using an EASY-nLC 1200 system (Thermo Scientific, Waltham, USA) equipped with an Acclaim PepMap RSLC RP C18 column (50 µm × 150 mm, 2 µm; Thermo Scientific, Waltham, USA) and a Q Exactive Plus Orbitrap mass spectrometer (Thermo Scientific, Germany). The flow rate was maintained at 350 nL min^-1^ with a linear gradient from 2% to 25% solvent B over 88 min, followed by an increase to 55% solvent B over 60 min. Solvent A was H₂O with 0.1% formic acid, and solvent B was 80% acetonitrile in H₂O with 0.1% formic acid. The Orbitrap operated in data-dependent acquisition mode, acquiring peptide signals from 385–1250 m/z at 70K resolution, with a maximum injection time of 75 ms and an AGC target of 3*10^6^ over 175 min. The top 10 precursor ions were isolated using a 2.5 m/z window and fragmented at a normalized collision energy (NCE) of 28. Fragment ions were acquired at 17.5K resolution, with a maximum injection time of 100 ms and an AGC target of 5e5. Singly charged ions, unassigned charges, and ions with charges >6 were excluded from fragmentation. Dynamic exclusion was set to 30 s, with peptide match preference enabled.

**Supplementary Figure 1. Correlation between OD_600_ and broth biomass concentration.** To determine this correlation, samples from the bioreactor broth were routinely taken during the D- and L-lactate bioreactor runs. The cell dry weight was determined, and the corresponding biomass concentration was correlated to the measured OD_600_ at the time of sampling. The correlation coefficient was used to convert OD measurements to biomass concentrations.


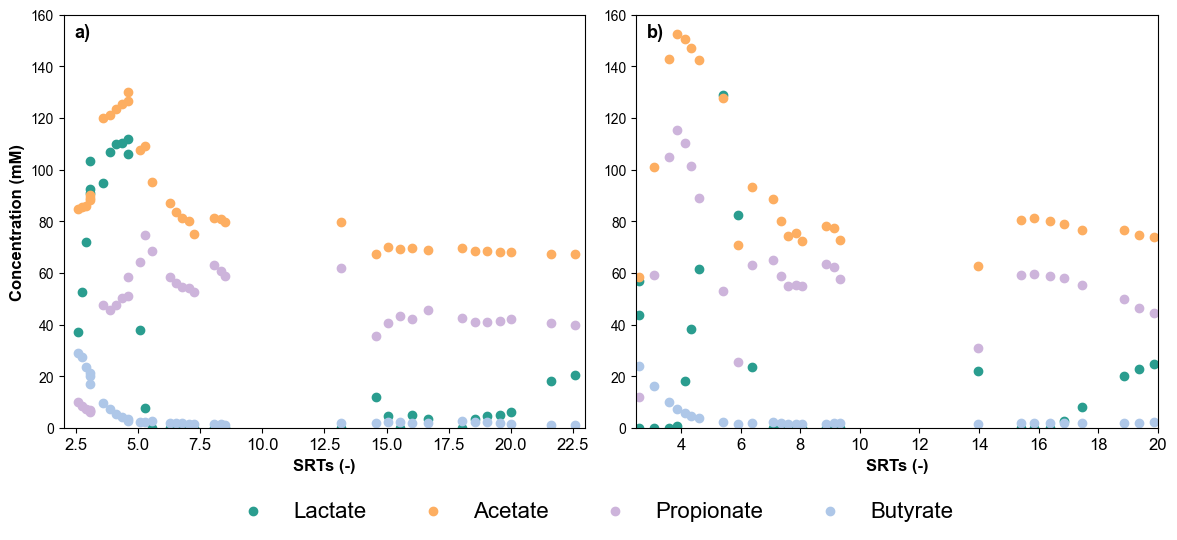


**Supplementary Figure 2. Product spectrum development in Experiment I: DL-lactate enrichment.** Panel a.) shows the product spectrum in biological replicate I. The initial start-up phase of the bioreactor was omitted from the graph. From 2.6 SRTs, the biological replicate bioreactor II, shown in panel b.), was inoculated from the effluent of replicate I. From 2.5 – 5 SRTs, the bioreactors were operated with double the amount of lactate and acetate in the feed due to a calculation error during media preparation. Initially, competition between butyrate producers and acetate/propionate producers took place, but the butyrate producers were outcompeted relatively early in the enrichment process. Due to a technical problem, a pH shock occurred after 5.5 SRTs in replicate II, after which the system required recovery. This prevented the SRTs from overlapping between replicates. The steady state at D = 0.01 h^-1^ was assumed between 5.5 and 8.5 SRTs in replicate I and 7.3 to 9.3 SRTs in replicate II, after which the dilution rate was increased to D = 0.02 h^-1^. Between 8.5 and 13 SRTs (replicate I), or 9.3 and 14 SRTs (replicate II), the bioreactor was operated without sampling. At D = 0.02 h^-1^, steady-state conditions could not be maintained because lactate concentrations increasingly accumulated.

**Supplementary Figure 3. Relative abundance of the metagenomics-identified taxa in the metagenome and metaproteome.** a.) Abundance heatmap of the species identified using shotgun metagenomics. The relative abundance (%) of the species in the bioreactor samples is displayed, along with the clustering of the samples. D: sample from D-lactate-fed chemostat, L: sample from D-lactate-fed chemostat, LtoD: samples from L-lactate-fed chemostat after switching to only D-lactate feeding and a recovery period, DtoL: samples from D-lactate-fed chemostat after switching to only L-lactate feeding and a recovery period. b.) Relative abundance of each genome based on protein abundance in the metaproteome. The bar plot shows the average relative abundance of the taxa in the LtoD (left) and DtoL (right) samples. MAGs representing less than 1% of the metaproteome, except *Propionibacterium freudenreichii*, were grouped and are shown as ‘Others’ in the bar plot.


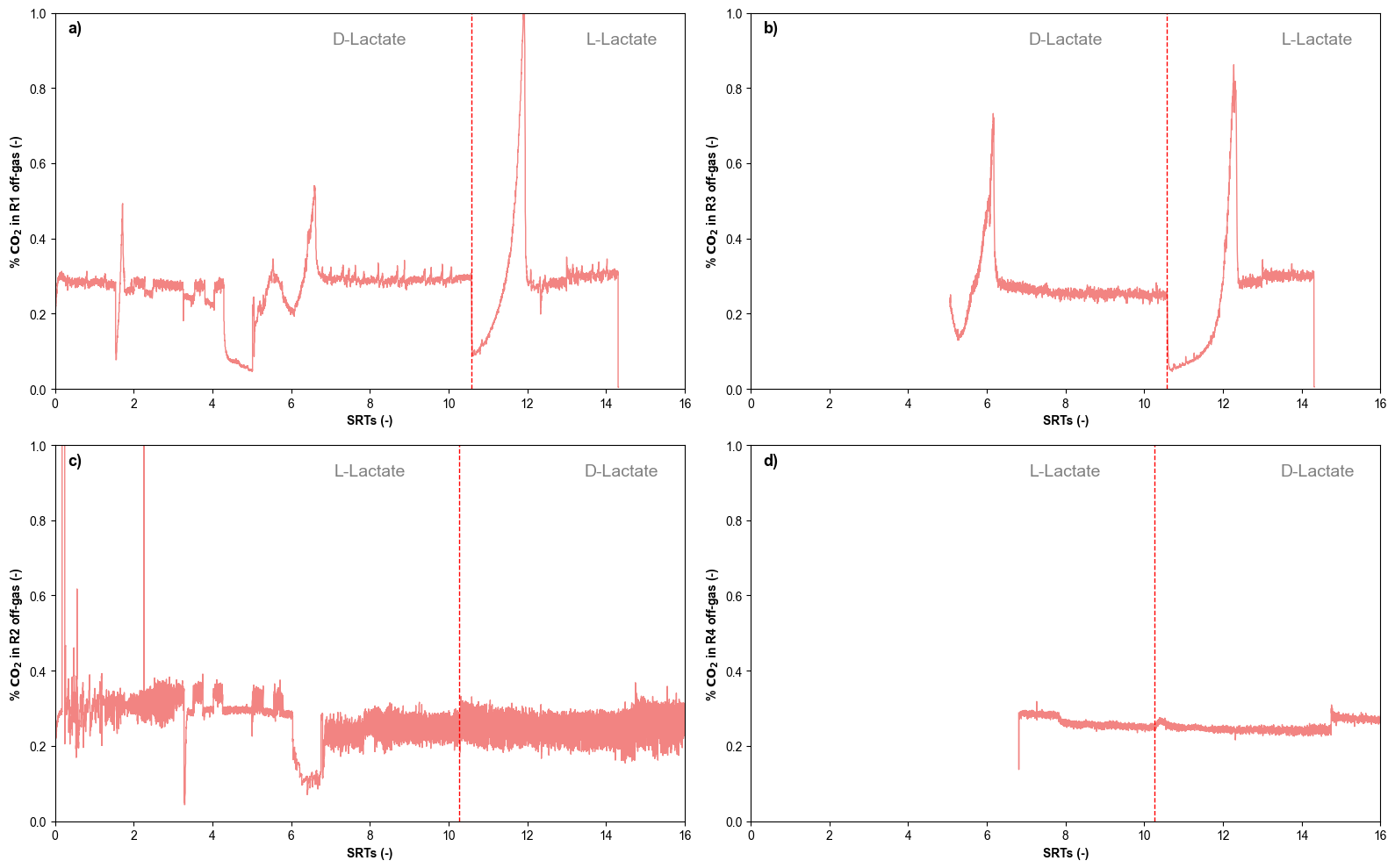


**Supplementary Figure 4. Full CO_2_ off-gas profiles during Experiment II.** CO_2_ profiles of all single-isomer-fed bioreactors over the entire operating period. Panels a.) and b.) show gas data of the bioreactors initially fed with D-lactate, while panels c.) and d.) show gas data of the bioreactors initially fed with L-lactate. At each measurement time, the gas stream composition was measured five times. Only the fifth measurement point was considered for these visualizations to ensure the measurement had stabilized. Off-gas measurements of bioreactors 3 and 4 (panels b. and d.) were started later during the operating period due to hardware limitations. There was consistently more noise on the channel of bioreactor 2 (panel c.), likely due to a small leak in the stream selector compartment of the mass spectrometer, causing minimal amounts of gas from other bioreactors in the lab to enter the channel. As the average concentration of CO_2_ and N_2_ matched the concentrations measured for the other biological replicate (panel d) and was within the same range as all other bioreactors, the data were considered reliable.


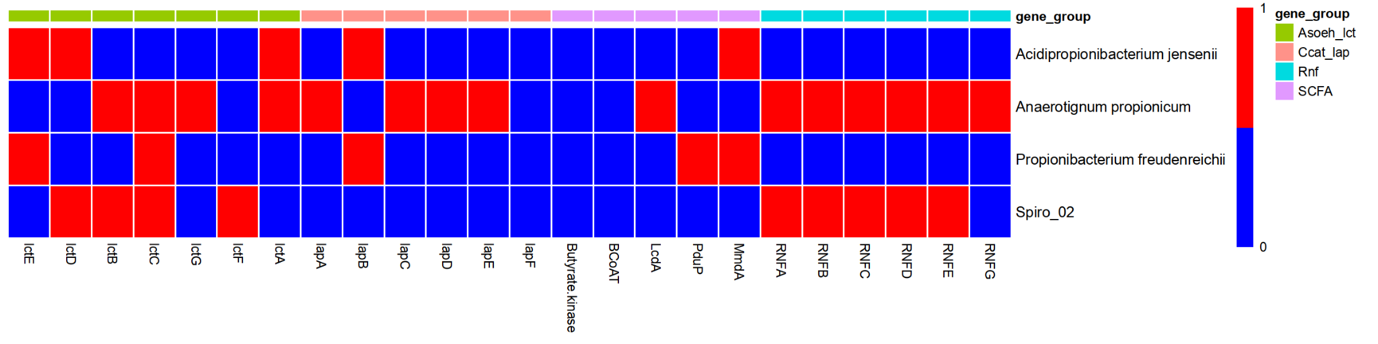
**Supplementary Figure 5. Presence of gene groups associated with lactate utilization in the selected bacterial genomes.** The genes were detected based on Diamond alignment with various thresholds of identity. Gene clusters included in the analysis were chosen according to Sheridan et al.^10^ and comprised the *lct* cluster from *Anaerobutyricum soehngenii* (Asoeh_lct), the *lap* cluster from *Coprococcus catus* (Ccap_lap), Rnf complex genes (Rnf) and short-chain fatty acid production genes (SCFA). Full names of genes indicated by short-hand gene nomenclature are as follows: lactate permease (*lctE*), NAD-independent lactate dehydrogenase (*lctD*), electron transfer flavoprotein subunit beta (*lctB*), electron transfer flavoprotein subunit alpha (*lctC*), acyl-CoA dehydrogenase (*lctG*), lactate racemase (*lctF*), LutR transcriptional regulator (*lctA*), propionyl CoA transferase (*lapA*), lactoyl CoA epimerase (*lapB*), lactoyl CoA dehydratase subunits (*lapC, lapD, lapE*), lactate permease (*lapF*), butyrate:CoA transferase (*BCoAT*), lactoyl-CoA dehydratase subunit alpha (*LcdA*), CoA-dependent propionaldehyde dehydrogenase (*PduP*), methylmalonyl-CoA decarboxylase subunit alpha (*MmdAI*)*,* and Rnf complex subunits (*RNFA – G*).


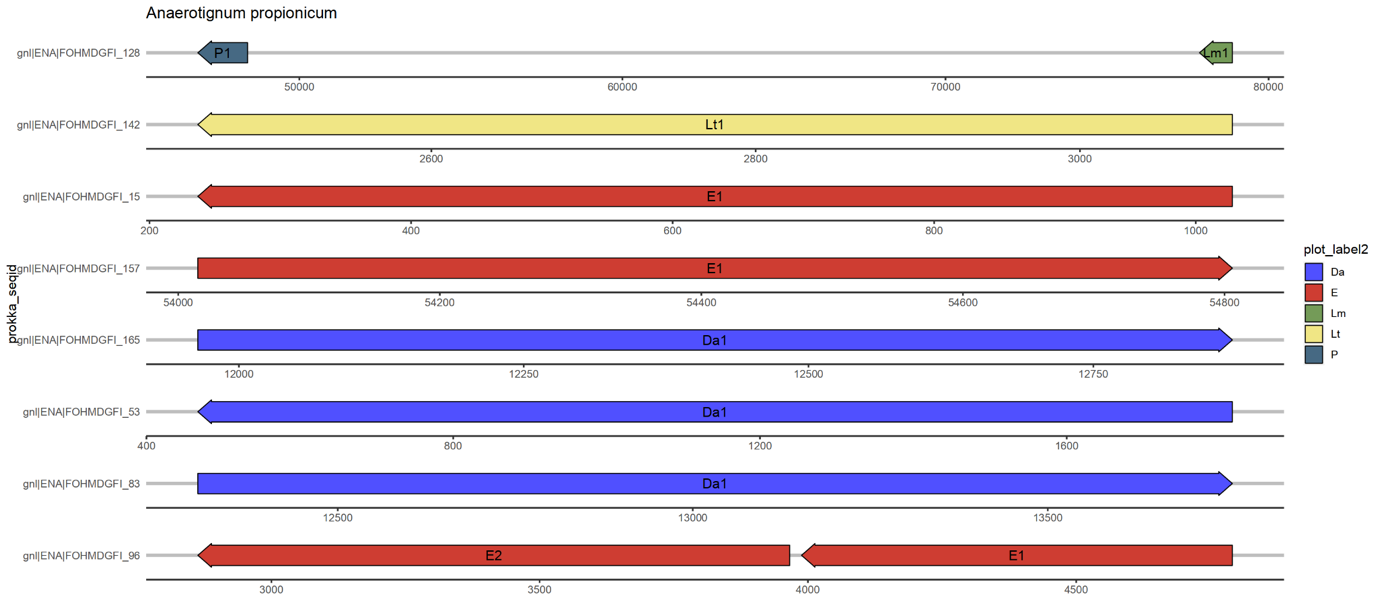

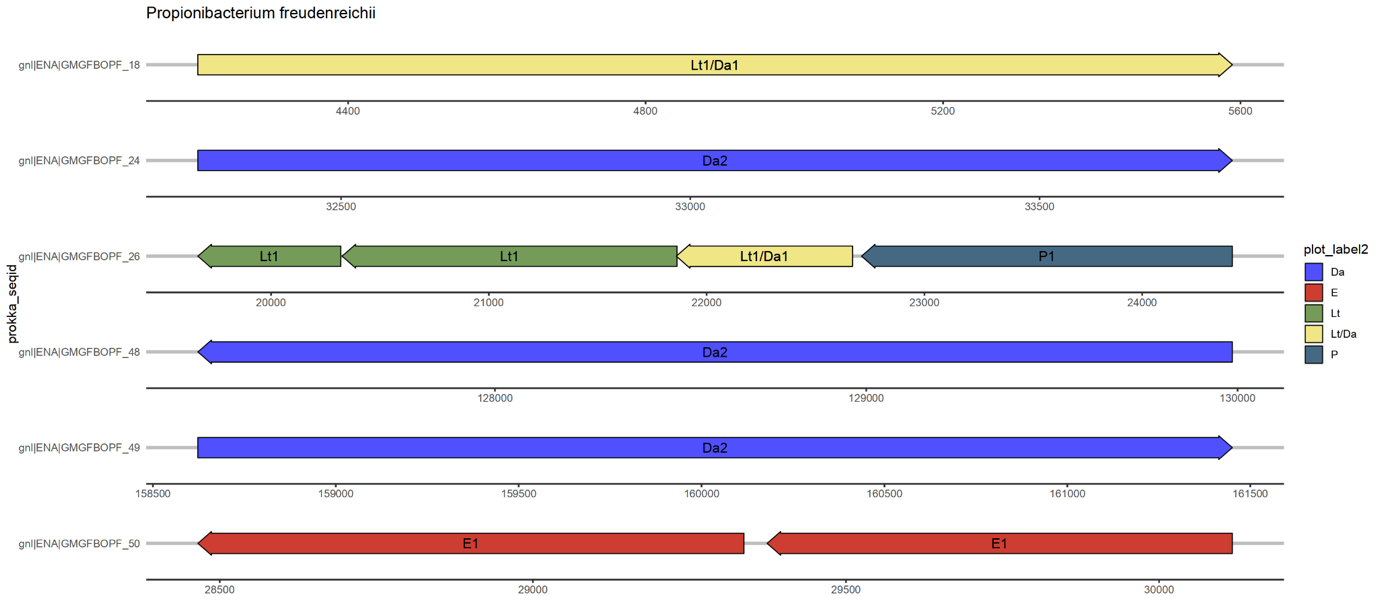

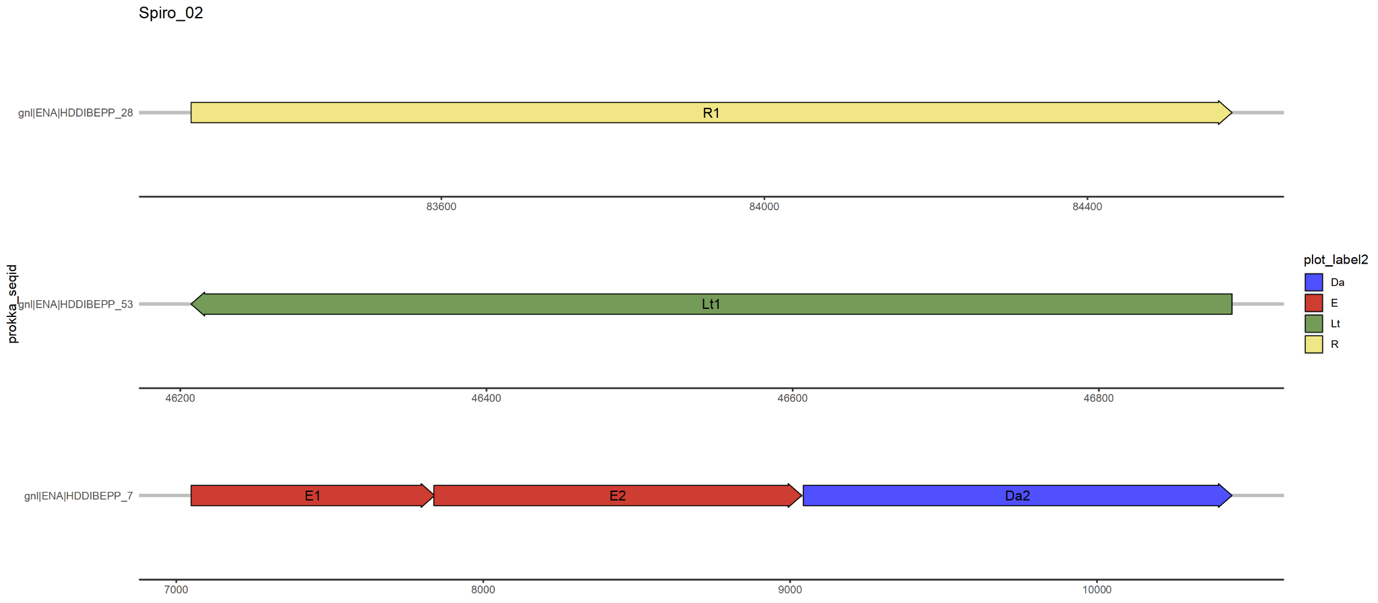

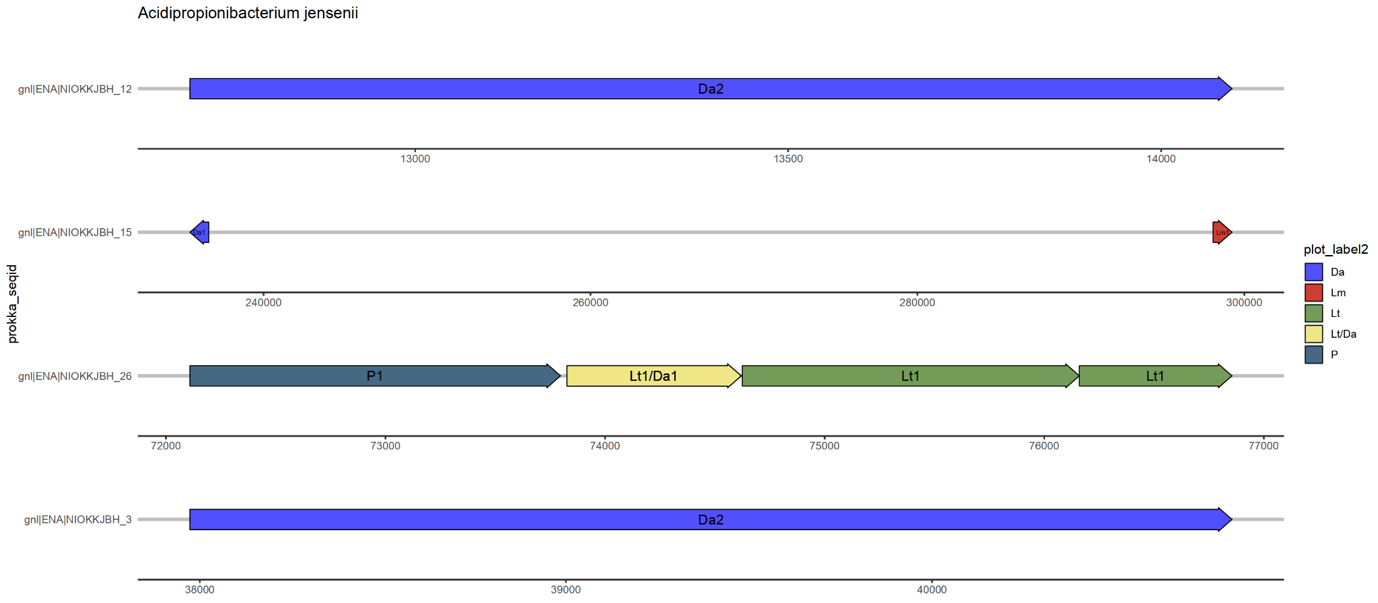


**Supplementary Figure 6. Genomic organization of lactate utilization loci.** Shown are the genes in which at least one marker functional domain of at least a lactate-utilization gene group was detected *via* hmmsearch. The labels display the name of the detected gene groups, followed by the number of respective detected domains. In cases where multiple groups are detected in a gene, these are concatenated, separated by a forward slash (“/”). Gene groups:

Lt - three-component L-lactate hydrogenase (PF02754, PF02589, PF13183, PF11870).

Da - FAD-dependent D-lactate dehydrogenase (PF01565, PF02913, PF09330, PF12838, PF02754).

Lm - FMN-dependent L-lactate dehydrogenase (PF01070, PF00173).

P - lactate permease (PF02652).

R - lactate racemase (PF09861).

E - ETF proteins alpha, beta (PF00766, PF01012).

**Supplementary Figure 7. Inoculum community composition at phylum level.** The community composition was determined by 16S rRNA gene amplicon sequencing.

**References**

1. Akbuğa-Schön T, Suzuki TA, Jakob D, Vu DL, Waters JL, Ley RE. The keystone gut species Christensenella minuta boosts gut microbial biomass and voluntary physical activity in mice. mBio. 2024;15. https://doi.org/10.1128/mbio.02836-23.

2. Goodrich JK, Waters JL, Poole AC, Sutter JL, Koren O, Blekhman R, et al. Human genetics shape the gut microbiome. Cell. 2014;159:789–99. https://doi.org/10.1016/j.cell.2014.09.053.

3. Zhernakova A, Kurilshikov A, Jan Bonder M, Tigchelaar EF, Schirmer M, Vatanen T, et al. Population-based metagenomics analysis reveals markers for gut microbiome composition and diversity. Science (1979). 2016;352:21.

4. Martin M. Cutadapt removes adapter sequences from high-throughput sequencing reads. EMBnet J. 2011;17. https://doi.org/https://doi.org/10.14806/ej.17.1.200.

5. Magoč T, Salzberg SL. FLASH: Fast length adjustment of short reads to improve genome assemblies. Bioinformatics. 2011;27:2957–63. https://doi.org/10.1093/bioinformatics/btr507.

6. Chen S, Zhou Y, Chen Y, Gu J. Fastp: An ultra-fast all-in-one FASTQ preprocessor. In: Bioinformatics. Oxford University Press; 2018. p. i884–90. https://doi.org/10.1093/bioinformatics/bty560.

7. Rognes T, Flouri T, Nichols B, Quince C, Mahé F. VSEARCH: A versatile open source tool for metagenomics. PeerJ. 2016;2016. https://doi.org/10.7717/peerj.2584.

8. Quast C, Pruesse E, Yilmaz P, Gerken J, Schweer T, Yarza P, et al. The SILVA ribosomal RNA gene database project: Improved data processing and web-based tools. Nucleic Acids Res. 2013;41. https://doi.org/10.1093/nar/gks1219.

9. Callahan BJ, McMurdie PJ, Rosen MJ, Han AW, Johnson AJA, Holmes SP. DADA2: High-resolution sample inference from Illumina amplicon data. Nat Methods. 2016;13:581–3. https://doi.org/10.1038/nmeth.3869.

10. Guerrini CJ, Botkin JR, McGuire AL. Reproducible, interactive, scalable and extensible microbiome data science using QIIME 2. Nat Biotechnol. 2019;37:850–2. https://doi.org/10.1038/s41587-019-0190-3.

11. Suzuki TA, Akbuğa-Schön T, Waters JL, Jakob D, Vu DL, Ballinger MA, et al. Selection and transmission of the gut microbiome alone shifts mammalian behavior. 2025. https://doi.org/10.1101/2025.01.21.634013.

12. Seemann T. Prokka: Rapid prokaryotic genome annotation. Bioinformatics. 2014;30:2068–9. https://doi.org/10.1093/bioinformatics/btu153.

13. Cantalapiedra CP, Hern̗andez-Plaza A, Letunic I, Bork P, Huerta-Cepas J. eggNOG-mapper v2: Functional Annotation, Orthology Assignments, and Domain Prediction at the Metagenomic Scale. Mol Biol Evol. 2021;38:5825–9. https://doi.org/10.1093/molbev/msab293.

14. Olm MR, Brown CT, Brooks B, Banfield JF. DRep: A tool for fast and accurate genomic comparisons that enables improved genome recovery from metagenomes through de-replication. ISME Journal. 2017;11:2864–8. https://doi.org/10.1038/ismej.2017.126.

15. Breitwieser FP, Baker DN, Salzberg SL. KrakenUniq: Confident and fast metagenomics classification using unique k-mer counts. Genome Biol. 2018;19. https://doi.org/10.1186/s13059-018-1568-0.

16. Olson RD, Assaf R, Brettin T, Conrad N, Cucinell C, Davis JJ, et al. Introducing the Bacterial and Viral Bioinformatics Resource Center (BV-BRC): a resource combining PATRIC, IRD and ViPR. Nucleic Acids Res. 2023;51 1 D:D678–89. https://doi.org/10.1093/nar/gkac1003.

17. Li W, O’Neill KR, Haft DH, Dicuccio M, Chetvernin V, Badretdin A, et al. RefSeq: Expanding the Prokaryotic Genome Annotation Pipeline reach with protein family model curation. Nucleic Acids Res. 2021;49:D1020–8. https://doi.org/10.1093/nar/gkaa1105.

18. Sheridan PO, Louis P, Tsompanidou E, Shaw S, Harmsen HJ, Duncan SH, et al. Distribution, organization and expression of genes concerned with anaerobic lactate utilization in human intestinal bacteria. Microb Genom. 2022;8. https://doi.org/10.1099/mgen.0.000739.

19. Buchfink B, Reuter K, Drost HG. Sensitive protein alignments at tree-of-life scale using DIAMOND. Nat Methods. 2021;18:366–8. https://doi.org/10.1038/s41592-021-01101-x.
